# Serum Omentin-1 and CS846: Biomarkers for diagnosis and post-TKA functional outcomes in knee osteoarthritis

**DOI:** 10.5937/jomb0-62196

**Published:** 2026-05-22

**Authors:** Yingzhao Qi, Zhixin Liu, Fengli Sun, Ran Liu, Jianfeng Deng, Longfei Hao, Jiayu Wang, Juyuan Gu

**Affiliations:** 1 The First Hospital of Qinhuangdao, Department of Orthopaedics, Qinhuangdao, Hebei, China; 2 The First Hospital of Qinhuangdao, Department of Orthopaedics, Qinhuangdao, The First Hospital of Qinhuangdao, Department of Orthopaedics, Qinhuangdao, Hebei, China; 3 The First Hospital of Qinhuangdao, Department of Orthopaedics, The First Hospital of Qinhuangdao, Department of Orthopaedics, Qinhuangdao, Hebei, China; 4 Hebei Medical University, Graduate School, Shijiazhuang, Hebei, China; 5 Hebei Medical University, Department of Joint, Shijiazhuang, Hebei, China

**Keywords:** omentum-1, CS846, knee osteoarthritis, total knee arthroplasty, diagnostic and prognostic biomarkers, omentin-1, CS846, osteoartritis kolena, totalna artroplastika kolena, dijagnostički i prognostički biomarkeri

## Abstract

**Background:**

To determine the synergistic diagnostic value of serum Omentin-1 and cartilage oligomeric matrix protein epitope CS846 (CS846) in knee osteoarthritis (KOA) and to explore their link to functional recovery following total knee arthroplasty (TKA), thereby contributing to improved early screening and prognostic strategies.

**Methods:**

Osmislili smo prospektivnu kohortnu studiju koja je obuhvatila 84 ispitanika (42 sa KOA i 42 zdrave kontrole). Tehnika enzimski povezanog imunosorbentnog testa (ELISA) korisćena je za merenje serumskog omentina-1, CS846 i inflamatornih indeksa (hs-CRP i ESR). ROC krive (Receiver Operating Characteristic) odredile su dijagno-stičku efikasnost, a za kombinovanu dijagnozu je uspostav-ljen logistički regresioni model. Funkcionalni ishodi, mereni WOMAC rezultatima, i status rehabilitacije su pregledani 6 meseci nakon operacije.

**Results:**

Analysis revealed reduced Omentin-1 and elevated CS846 in KOA patients versus controls. The diagnostic per-formance (AU C= 0.851) of the biomarker combination surpassed that of individual tests. Patients with KL1-2 grades exhibited elevated Omentin-1 but lower CS846 levels compared to those with KL3-4 grades (P&lt; 0.05). An inverse correlation was observed between Omentin-1 and inflammatory markers (hs-CRP ESR), contrasting with the positive correlation for CS846. Post-treatment, poor recov-ery was associated with more pronounced decreases in Omentin-1 and increases in CS846 (P&lt; 0.05). The COM-bined model effectively predicted suboptimal rehabilitation outcomes (AU C= 0.857).

**Conclusions:**

The synergistic use of serum Omentin-1 and CS846 biomarkers boosts early detection capability for KOA while accurately forecasting post-TKA functional out-comes.

## Abstract (sr)

**Uvod:**

Cilj je bio da se utvrdi sinergistička dijagnostička vrednost serumskog Omentina-1 i epitopa CS846 (CS846) oligomernog matriksnog proteina hrskavice kod osteo-artritisa kolena (KOA) i da se istraži njihova veza sa funkcio-nalnim oporavkom nakon totalne artroplastike kolena (TKA), čime bi se doprinelo poboljsanju ranog skrininga i prognostičkih strategija.

**Metode:**

Sprovedena je retrospektivna analiza koja je obu-hvatila 90 pacijentkinja sa PE i 90 zdravih trudnica. Ru-tinski hematoloski i biohemijski parametri mereni su koris-ćenjem automatizovanog hematoloskog analizatora, odnosno automatizovanog biohemijskog analizatora, i izra-čunati su izvedeni indeksi (NLR, NHR, MHR, SII). Neza-visni faktori rizika određeni su multivarijantnom logističkom regresijom, a prediktivni model je validiran analizom ROC krive (recimber operating characteristic).

**Rezultati:**

Analiza je pokazala smanjen Omentin-1 i povisen CS846 kod pacijenata sa KOA u odnosu na kontrolnu grupu. Dijagnostičke performanse (AUC=0,851) kombinacije bio-markera su premasile one pojedinačnih testova. Pacijenti sa KL1-2 stepenom pokazali su povisen Omentin-1, ali niže nivoe CS846 u poređenju sa onima sa KL3-4 stepenom (P&lt; 0,05). Inverzna korelacija je primećena između Omen-tina-1 i inflamatornih markera (hs-CRP ESR), sto je suprotno pozitivnoj korelaciji za CS846. Nakon tretmana, los oporavak je bio povezan sa izraženijim smanjenjem Omentina-1 i povećanjem CS846 (P&lt; 0,05). Kombinovani model je efikasno predvideo suboptimalne ishode rehabilitacije (AUC=0,857).

**Zaključak:**

Sinergistička upotreba serumskih biomarkeraOmentin-1 i CS846 povecava mogucnost ranog otkrivanjaakutne aorte, uz precizno predviđanje funkcionalnih ishodanakon totalne endoproteze kolena (TKA).

## Introduction

As a leading degenerative joint disease globally, knee osteoarthritis (KOA) manifests through progressive cartilage degradation, synovitis, and osteophyte formation. A disability rate of 10%-15% is reported among the predominantly middle-aged and elderly population affected [1]. Early-stage KOA, characterized by non-specific symptoms, is frequently occult on conventional radiography (X-ray) since the modality is insufficient for identifying cartilage loss below 30% [2]. The use of magnetic resonance imaging (MRI), despite its greater sensitivity, is constrained by high costs and limited accessibility, driving the need for accessible, specific biomarkers to aid early diagnosis and disease monitoring [3]. In cases of advanced KOA, total knee arthroplasty (TKA) is the sole definitive treatment; however, approximately 15%-20% of recipients experience persistent issues such as stiffness and pain, emphasizing the need for better predictors of TKA outcomes [4].

Biomarker research in KOA centers on inflammatory mediators and cartilage metabolic byproducts [5][6]. The adipokine Omentin-1, secreted primarily from visceral adipose tissue, has dual anti-inflammatory and cartilage-protective roles, including promoting chondrocyte proliferation and inhibiting apoptosis [7]. Recent evidence confirms an inverse correlation between circulating Omentin-1 levels and cartilage damage severity in KOA [8]. Cartilage oligomeric matrix protein epitope CS846 (CS846), a glycosylated metabolite of cartilage oligomeric matrix protein, is a specific marker of cartilage matrix synthesis, capturing the compensatory production of cartilage components in early KOA [9]. Omentin-1 may potentially support CS846-driven repair indirectly, though this requires validation via mechanistic studies. Conversely, CS846-reflected cartilage synthesis could be modulated by the Omentin-1-influenced metabolic milieu. However, most studies investigate Omentin-1 and CS846 independently. Their cooperative dynamics and joint diagnostic utility in KOA therefore remain unestablished.

For the first time, this research comprehensively assesses the utility of combining serum Omentin-1 and CS846 for diagnosing early-stage KOA. Additionally, we examine whether these markers correlate with knee function at 6 months following TKA and whether they exhibit synergistic interactions. Should these biomarkers prove valuable for early detection, they may fill a critical gap in current diagnostic methodologies. Additionally, establishing a link with postoperative functional outcomes may inform preoperative evaluation and rehabilitation planning. These findings have the potential to enhance the early biomarker framework for KOA and provide a theoretical basis for improving long-term patient management after TKA.

## Materials and methods

### Sample size estimation

To determine the necessary sample size, we referred to an earlier investigation [10] that observed an effect size (Cohen's d ≈ 1.2) in serum Omentin-1 concentrations when comparing healthy individuals to those diagnosed with early-stage KOA. Power analysis conducted with PASS 15.0, assuming α = 0.05 (two-tailed) and 80% statistical power, indicated that each group required at least 38 participants. Considering a 10% dropout rate, 42 KOA patients and 42 gender- and age-matched healthy controls were finally included, totaling 84 participants.

### Patient selection

Study design: The research employed a single-center, prospective cohort design spanning the period from January 2024 to February 2025. Participant recruitment: Participants were sourced from the orthopedic outpatient clinic and inpatient wards of our hospital after rigorous screening. General inclusion criteria included: age ranging from 45 to 75 years, irrespective of sex; and voluntary participation confirmed by written informed consent. Eligibility criteria for the healthy control group: individually matched to the KOA group for age (difference 5 years) and sex (1:1 ratio); no history of knee pain, swelling, or functional impairment; Kellgren-Lawrence (KL) grade 0 on standing anteroposterior and lateral knee radiographs; normal serum hs-CRP and ESR levels. Eligibility criteria for the KOA group: fulfilling the established diagnostic criteria for KOA [11]. Exclusion criteria (for all participants): other arthritic conditions (e.g., rheumatoid arthritis, gout, psoriatic arthritis); history of knee trauma, infection, or neoplasia; significant comorbid systemic diseases affecting the heart, liver, or kidneys; recent (within 3 months) intake of drugs known to influence cartilage turnover or inflammatory responses; Pregnancy or lactation; inability to complete questionnaires or follow-up (e.g., due to cognitive impairment or remote residence). This study received approval from our hospital's Ethics Committee and was performed with strict adherence to the tenets of the Declaration of Helsinki. Following matching, the two groups were balanced in terms of age, gender, and family history of KOA, with no statistically significant differences found (P>0.05). All enrolled KOA patients underwent TKA as the definitive treatment, and no non-surgical patients were included in the study.

### Treatment and follow-up

All KOA patients received TKA performed by a consistent surgical team. They were followed up for at least six months postoperatively, including monthly evaluations and scheduled re-examinations. Recovery status was defined as favorable if patients could walk unaided for >30 minutes without pain and had a WOMAC total score <20 (based on established thresholds); otherwise, recovery was deemed suboptimal.

### Detection methods

Peripheral blood samples (3 mL) were obtained from fasting participants, both upon enrollment and after therapy at discharge. Serum was isolated by centrifugation under refrigeration (3000 X g, 10 min, 4°C), divided into aliquots, and cryopreserved at -80°C until analysis. Omentin-1 and CS846 measurements were performed using enzyme-linked immunosorbent assay (ELISA). All kits were purchased from Shanghai Kanglong Biotechnology Co., LTD. (China). The protocol comprised: generating a dilution series of standards; incubating standards/samples (100 μL) in precoated plates (2 h); sequential incubations with biotin-conjugated antibody (100 μL / well, 1 hour at 37°C) and horseradish peroxidase-labeled streptavidin (100 μL / well, 30 minutes at room temperature); and terminating the chromogenic reaction before reading the optical density at 450 nm. Analyte concentrations were derived from standard curves and multiplied by dilution factors. For quality control, Bio-Rad Lyphochek Immunoassay Plus control serum (Lot No. 604UN) was used daily at low, medium, and high levels, accepting only results with a coefficient of variation (Cv ) under 10%.

Additionally, both groups underwent testing for hs-CRP and ESR upon admission. Hs-CRP quantification was conducted on a Roche Cobas c702 automated biochemical analyzer (USA). Serum samples were analyzed immediately after separation. The assay involved sequential addition of 10 μL sample, 240 μL buffer, and 50 μL latex particle reagent into reaction cuvettes. Following a 5-minute incubation at 37°C, absorbance variations at 570 nm were measured, and hs-CRP concentrations were derived from turbidity data. Daily quality assurance utilized Roche Precinorm U and Precipath U control materials, with a CV threshold of 3%. ESR testing was carried out on an Alifax Test 1 automated instrument (Italy). Venous blood (2 mL) was drawn into EDTA-K2 tubes and mixed immediately. The anticoagulated blood was then loaded into an ESR tube to the 100 mm mark and positioned vertically in the device, which automatically reported the ESR in mm / h. Quality control procedures included daily testing with Alifax Control Line (Batch No. CL-03), accepting CV values under 5%.

Laboratory technicians were blinded to the group allocation (KOA *vs.* control) during sample analysis to minimize testing bias.

### Statistical analysis

Analyses were performed in SPSS 34.0. Qualitative variables like sex and KOA family history are shown as n (%) and analyzed with the chi-square test. For quantitative measures such as Omentin-1 and CS846, normality was verified with the Shapiro-Wilk test, with the data summarized as (x̄±s). Independent and paired t-tests were used for between- and within-group comparisons, respectively. ROC analysis evaluated diagnostic power, and logistic regression modeled combined detection. Relationships were quantified via Pearson correlation. For multiple correlation analyses (e.g., Omentin-1/CS846 with inflammatory markers), a Bonferroni correction was applied to adjust the significance level (P < 0.025 for two tests). Significance was set at P<0.05.

## Results

### Diagnostic potential of Omentin-1 and CS846 in KOA

**Figure 1:**
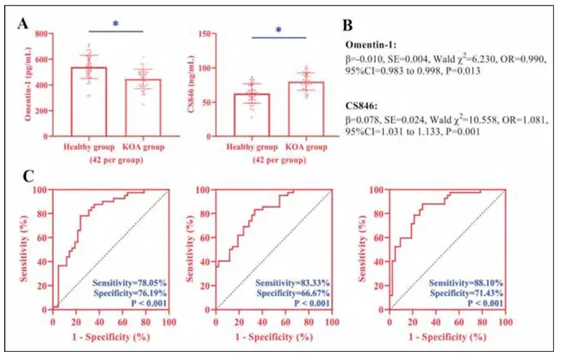
Diagnostic efficacy of Omentin-1,CS846 for KOA. (A) Comparison of Omentin-1 and CS846 between KOA group and healthy group. (B) Logistic regression of the effects of Omentin-1 and CS846 on KOA. (C) ROC curve of Omentin-1 and CS846 for the diagnosis of KOA (Omentin-1: AUC=0.800, CS846: AUC=0.812, Omentin-1+CS846: AUC=0.851). * indicates that the difference between groups is statistically significant (P < 0.05).

In KOA, serum Omentin-1 was significantly decreased and CS846 was elevated compared to healthy controls (P<0.05). Individually, Omentin-1 and CS846 demonstrated diagnostic potential for KOA, with AUC values of 0.800 (95%CI: 0.705-0.898) and 0.812 (95%CI: 0.724-0.901), respectively. Regression analysis identified both markers as independent predictors of KOA (P<0.05). A combined diagnostic model, constructed based on regression coefficients ( ), showed improved diagnostic performance (AUC=0.851, 95%CI: 0.770-0.932) over individual tests, achieving a sensitivity of 88.10% and a specificity of 71.43% (P<0.05) (Figure 1).

### Association of Omentin-1 and CS846 with disease progression in KOA

**Figure 2:**
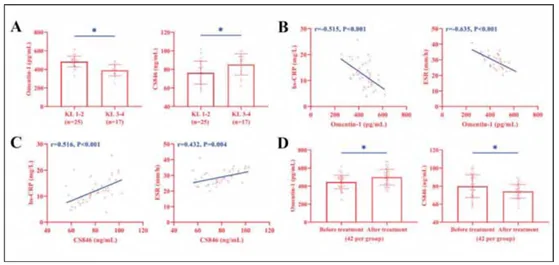
Association of Omentin-1,CS846 and KOA progression. (A) Comparison of Omentin-1 and CS846 between KL 1-2 and KL 3-4 patients. (B) The correlation of Omentin-1 with hs-CRP and ESR. (C) The correlation between CS846 and hs-CRP ESR. (D) Comparison of Omentin-1 and CS846 before and after treatment. * indicates that the difference between groups is statistically significant (P < 0.05).

Patients diagnosed with early-stage KOA (KL grades 1-2) had higher Omentin-1 and lower CS846 concentrations than those with severe KOA (KL grades 3-4) (P<0.05). According to correlation analysis, Omentin-1 showed negative correlations with inflammatory markers hs-CRP and ESR (P<0.05), while CS846 was positively correlated with both (P<0.05). Post-treatment, Omentin-1 rose by 12.05% (44.6.59±74.99 pg/mL vs. 500.40±86.78 pg/mL), and CS846 declined by 7.18% (79.97±12.68 ng/mL vs. 74.23±7.57 ng/mL) (P<0.05) (Figure 2).

### Follow-up outcomes

**Figure 3:**
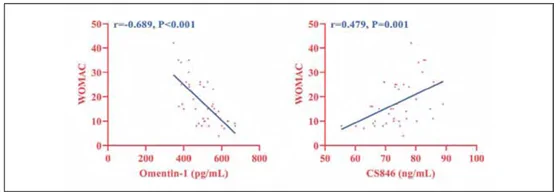
The correlation of Omentin-1, CS846 and WOMAC score (scale 0-96, higher scores indicate worse function) after treatment.

All enrolled KOA patients completed follow-up for a minimum of six months (median: [10 (8, 12)] months). Recovery was satisfactory in 28 patients and suboptimal in 14. At the six-month assessment, the WOMAC total score (17.62±9.26) showed a significant inverse association with Omentin-1 and a positive relationship with CS846 (P<0.05) (Figure 3).

### Utility of Omentin-1 and CS846 in assessing post-TKA rehabilitation efficacy

**Figure 4:**
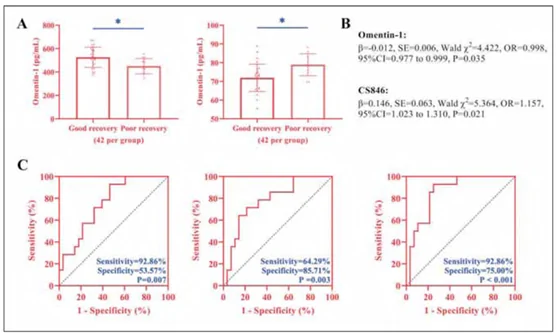
Predictive effect of Omentin-1,CS846 on poor rehabilitation after TKA. (A) Comparison of Omentin-1,CS846 between patients with good and poor recovery. (B) ROC curve analysis of Omentin-1 and CS846 in predicting poor rehabilitation after TKA (Omentin-1: AUC=0.758, CS846: AUC=0.781, Omentin-1+CS846: AUC=0.857). * indicates that the difference between groups is statistically significant (P < 0.05).

Individuals with impaired recovery showed significantly decreased Omentin-1 and elevated CS846 relative to those recovering well (P<0.05). The AUC of Omentin-1 and CS846 for predicting poor recovery were 0.758 (95%CI: 0.613-0.902) and 0.781 (95%CI: 0.635-0.926), respectively. Both biomarkers were identified as independent predictors in regression models. When used in combination, Omentin-1 and CS846 effectively predicted suboptimal rehabilitation, achieving 92.86% sensitivity and 75.00% specificity, with an AUC of 0.857 (95%CI: 0.744-0.970), supporting its reference value in clinical practice (Figure 4).

## Discussion

Early diagnosis and unpredictable functional restoration after surgery remain major clinical concerns in KOA, the leading degenerative joint condition worldwide [12]. In this study, we systematically assessed, for the first time, the combined diagnostic value of serum Omentin-1 and CS846 in early-stage KOA and examined their association with post-TKA functional recovery. Data indicated substantially lowered Omentin-1 and raised CS846 concentrations among KOA patients. The dual-marker detection enhanced diagnostic accuracy, yielding an AUC of 0.851, along with 88.10% sensitivity and 71.43% specificity. Stratification by KL grade revealed that Omentin-1 was more abundant in KL1-2 cases relative to KL3-4, with CS846 demonstrating a reverse profile—implying their potential involvement in disease advancement. Additionally, individuals with suboptimal recovery following TKA displayed reduced Omentin-1 and augmented CS846. The combined model predicted poor rehabilitation outcomes with 92.86% sensitivity. The study thus proposes Omentin-1 and CS846 as a viable biomarker set to guide early screening and rehabilitation strategies in KOA.

Omentin-1, an anti-inflammatory adipokine, exerts protective effects by suppressing the NF-κB pathway, thereby reducing the production of pro-inflammatory cytokines such as IL-6 and TNF-α [13]. It also promotes chondrocyte proliferation and inhibits apoptosis [14]. This study found markedly reduced serum Omentin-1 levels in KOA patients, which correlated negatively with inflammatory markers hs-CRP and ESR. This suggests that lower Omentin-1 levels may serve as an indicator of ongoing synovitis. We also observed an inverse relationship between Omentin-1 expression and radiographic severity; levels were higher in KL1-2 patients than in KL3-4 cases. This indicates that the protective, anti-inflammatory influence of Omentin-1 wanes with advancing cartilage destruction. This aligns with established evidence linking lower Omentin-1 levels to greater cartilage damage [7]. Increased serum levels of CS846—a specific indicator of cartilage matrix synthesis— are generally associated with heightened cartilage repair [15]. Mechanistically, in the early phase of KOA, chondrocytes initiate a compensatory repair response characterized by increased synthesis of immature collagen types such as type II collagen. CS846, a glycosylated product of such collagens, rises accordingly, reflecting active matrix production [16]. As KOA advances, chondrocyte function deteriorates and apoptosis escalates, ultimately reducing CS846 synthesis and serum abundance. Our results further confirm this dynamic trend. Notably, an inverse dynamic relationship was observed between Omentin-1 and CS846 dynamics, hinting at their potential biological antagonism or synergy. Omentin-1's anti-inflammatory action might facilitate CS846-driven repair by reducing synovitis, while elevated CS846 may signal stressed chondrocyte compensation [17]. Clinically, combining both markers achieved a higher diagnostic AUC for KOA and post-TKA evaluation than single-marker testing, underscoring their complementary roles in reflecting the inflammation-repair imbalance. This supports the growing advocacy for »biomarker panels to optimize diagnostics« [18], positioning multi-parameter analysis as a key future strategy in KOA biomarker research.

Hence, simultaneous measurement of Omentin-1 and CS846 has potential utility in the early identification of KOA and in tracking disease status throughout therapeutic interventions. This strategy supports earlier postoperative risk classification and tailored rehabilitation. Meanwhile, reductions in Omentin-1 and abnormal rises in CS846 appear to correspond to an »anti-inflammatory impairment« and a »repair imbalance,« respectively, in the pathophysiology of KOA. Future directions might involve therapeutic delivery of Omentin-1 mimetics (e.g., recombinant forms) or targeting CS836 biosynthesis (e.g., using glycosylation modifiers) to improve long-term outcomes in affected patients.

However, the small sample size may limit the generalizability of AUC values and increase the risk of overfitting. Validation through larger, multi-center cohorts is urgently needed. Moreover, the scarcity of sampling time points means the lack of sequential pre- and post-operative data. Longer follow-up, extending beyond 12 months, could more fully reveal the connection between biomarker levels and functional recovery. Lastly, while the study suggests a possible interaction between Omentin-1 and CS846, the molecular mechanism has not been confirmed through in vitro or animal model experiments. Subsequent investigations ought to include basic experimental approaches to clarify their regulatory interplay. Furthermore, potential confounders such as BMI, body weight, and concomitant medications (e.g., anti-inflammatory drugs) that may influence Omentin-1 levels were not controlled, which could affect result generalizability.

## Conclusion

The combination of serum Omentin-1 and CS846 enhances early KOA diagnosis and predicts post-TKA functional outcomes, offering a novel strategy for personalized management. Future studies should validate these findings in larger cohorts and explore underlying mechanisms.

## Dodatak

### Funding

This study was supported by the S&T Program of Qinhuangdao (No.202201B021).

### Availability of data and materials

The data that support the findings of this study are available from the corresponding author upon reasonable request.

### Conflict of interest statement

All the authors declare that they have no conflict of interest in this work.
